# Brachial blood flow and pressure responses are unrelated to the greater isometric handgrip tolerance of females compared to males

**DOI:** 10.14814/phy2.70871

**Published:** 2026-04-15

**Authors:** João L. Marôco, Joseph Scangas, Megan Borges, Eric Bracken, Anton Pecha, Tracy Baynard, Bo Fernhall

**Affiliations:** ^1^ Integrative Human Physiology Laboratory, Manning College of Nursing & Health Sciences University of Massachusetts Boston Boston Massachusetts USA

**Keywords:** blood pressure, brachial blood flow, fatigue, isometric handgrip, sex differences

## Abstract

Isometric handgrip (IHG) training lowers blood pressure (BP), but sex differences in fatigability may be confounding. We hypothesized that the greater time‐to‐fatigue during IHG in females would relate to preserved brachial blood flow, with males exhibiting reductions in flow. Fifteen females and 14 males (18–32 years) completed a fatiguing IHG at 30% of maximum voluntary contraction (MVC). Brachial diameter and blood velocity were assessed with B‐mode and Doppler ultrasound, and BP via beat‐to‐beat finger photoplethysmography. Both sexes exhibited similar non‐significant increases in brachial flow during IHG (Δ_HG‐rest_ = 30 mL min^−1^, *p* = 0.200). Post‐exercise hyperemia was greater in males (Δ15_s_post‐rest = 182 mL min^−1^, 95% CI: 130–233 mL min^−1^, *p* < 0.001) than in females (Δ15_s_post‐rest = 89 mL min^−1^, 95% CI: 39–138 mL min^−1^, *p* < 0.001). Despite the mean arterial pressure response being greater in males (Δ = 30 mmHg, 95% CI: 24–37 mmHg, *p* < 0.001; vs. Δ = 17 mmHg, 95% CI: 10–23 mmHg, *p* < 0.001), conductance was similar between sexes during IHG (*p* = 0.470). Time‐to‐fatigue was greater in females (243‐s vs. 181‐s, *p* = 0.021) and unrelated to flow. Controlling for MVC, brachial diameter, or arm circumference did not affect results. Brachial flow and pressor responses were unrelated to the greater fatigue resistance of young females. The greater post‐exercise hyperemia in males suggests a greater ischemic stimulus, independent of blood flow.

## 1 INTRODUCTION

Isometric handgrip training is increasingly recognized as an anti‐hypertensive exercise modality, with such benefits suggested to be greater among healthy young adults than those with hypertension (Alves et al., 2022; Smart et al., 2019). Considering the rising prevalence of early‐onset hypertension (i.e., <35 years) (Siddiqi et al., 2024), blood pressure‐lowering effects of isometric handgrip training hold potential as a preventive tool. However, the well‐documented sex difference in fatigability to low‐intensity isometric exercise tasks, wherein females are more fatigue‐resistant than males (Hunter, 2014), may affect interpretation and use. Thus, investigating mechanisms behind sex dimorphism in isometric exercise fatigability is clinically relevant and may help reduce barriers to widespread use of isometric handgrip training.

Several mechanisms have been advanced to explain sex differences in isometric exercise fatigability, including dimorphism in the coordinated response of blood flow and pressure (BP), as well as skeletal muscle mass and strength (Hunter, 2016). These mechanisms are intertwined, as the smaller muscle mass and strength of females, compared to males, result in lower intramuscular pressures, which hypothetically reduce arterial compression and better preserve blood flow in females during isometric contractions (Barnes, 1980; Hunter, 2016; Thompson et al., 2007). While physiologically plausible, Thompson et al. (Thompson et al., 2007) reported no sex difference in the incremental response of brachial flow to low‐intensity isometric handgrip exercise (i.e., 20%MVC) when normalized to forearm volume, despite females exercising longer until task failure. However, vascular occlusion during isometric exercise appears only to impede flow when contracting intensities exceed ~20% MVC (Barnes, 1980). Since most isometric handgrip training protocols use intensities of 30%–40%MVC to balance sympathoexcitation with tolerability, it is important to determine whether brachial flow directed to working muscles follows the above‐reported pattern. Notably, Thompson et al. (2007). disregarded perfusion pressure responses (i.e., mean arterial pressure), and diameter changes during exercise as key determinants of blood flow. Compared to males, the smaller pressor response in females, linked in part to attenuated firing of the exercise pressor reflex, should theoretically reduce blood flow, but is inconsistently implicated with greater fatigue resistance of females to isometric exercise (Hunter & Enoka, 2001; Keller et al., 2022). It is plausible that greater activation of the exercise pressor reflex in males could contribute to reduced blood flow during fatiguing isometric exercise via exaggerated sympathetically mediated vasoconstriction as observed with hypertension (Barbosa et al., 2016; Greaney et al., 2015). Hence, it remains uncertain whether the flow and pressor responses contribute to the sex dimorphism in fatigability to isometric exercise.

Post‐exercise hyperemic responses have been implicated in sex differences in fatigability (Hunter & Enoka, 2001; Hunter et al., 2006, 2009; Kent‐Braun et al., 2002). Although males exhibit heightened ischemic stimuli during isometric exercise, which results in increased accumulation of vasoactive metabolites (e.g., adenosine, lactate) and greater post‐exercise hyperemia compared to females, this response fails to explain sex dimorphism in fatigability (Hunter et al., 2006, 2009). In these studies, blood flow was estimated using venous occlusion plethysmography, which lacks temporal resolution, alters hemodynamics via rapid cuff inflation/deflation cycles, and is inaccurate at high‐flow states (i.e., post‐exercise) (Chuah et al., 2004; Gliemann et al., 2018; Saltin, 2007). Thus, the use of Doppler ultrasound, the non‐invasive criterion in high‐flow states, is required to confirm these findings. Moreover, there is growing interest in assessing flow‐mediated dilation (FMD) in response to sustained shear stress from handgrip exercise, which may better reflect physiological states than transient post‐occlusion changes (Tremblay & Pyke, 2018). While sustained stimulus FMD is sensitive to chronic conditions (e.g., obesity) and acute (e.g., mental stress) stressors (Slattery et al., 2016; Szijgyarto et al., 2014), whether sex differences exist similar to traditional FMD remains unknown.

Therefore, this study tested the hypothesis that brachial blood flow would be preserved in young females but reduced in males during fatiguing isometric handgrip exercise (IHG) at 30% MVC, with these changes related to time‐to‐task failure. We also hypothesized that males would exhibit greater post‐exercise hyperemia and higher sustained FMD than females, with changes unrelated to time‐to‐task failure.

## 2 METHODS

### 2.1 Study design

The present investigation was designed as a cross‐sectional, repeated‐measures experiment. Participants reported to the laboratory in a 4‐h fasting state and refrained from coffee/caffeine, alcohol, and vigorous exercise for 12 h before the visit. To minimize diurnal variation, participants visited the laboratory in the mornings. After an initial 15‐min rest period, traditional FMD was evaluated. Following another 15‐min of rest, participants performed a fatiguing IHG bout, during which brachial blood flow and pressure were measured continuously and for 3‐min post‐exercise. The University of Massachusetts Boston Institutional Review Board approved the trial, with all testing procedures aligning with the Declaration of Helsinki.

### 2.2 Participants

Participant recruitment occurred via word‐of‐mouth referrals, institutional email broadcast, social media outreach from the University of Massachusetts Boston, and via flyers distributed throughout the Boston area. Twenty‐nine healthy young volunteers (15 females, 14 males, aged 18–32 years) participated. Eligible participants were those with (1) normal BP as per the American Heart Association (<130/90 mmHg) and ages between 18 and 40 years; (2) BMI <30 kg/m^2^; (3) no cardiovascular risk factors and clinically diagnosed cardiovascular disease; (4) recreationally active patterns (≤2 days of structured physical activity). Prospective participants were excluded if they used antihypertensive, vasoactive, or cardioactive medications; had obesity (BMI >30), hypertension, diabetes, depression or anxiety, long COVID, were smokers, or were pregnant or had menstrual cycle irregularities. All participants provided written informed consent.

### 2.3 Fatiguing isometric handgrip bout

MVCs were based on three attempts separated by 30‐s, with participants in the supine position. Participants were verbally encouraged to give a maximal effort. The highest MVC was used to define the isometric exercise workload at 30% MVC. Afterwards, all participants performed a fatiguing dominant‐handed isometric handgrip exercise using a digital handgrip transducer connected to a data acquisition system (Powerlab 16/35; ADInstruments, BellaVista, NSW, Australia). The handgrip transducer was adjusted to the hand size of each participant based on individual preference (i.e., the distance between handles). Fatigue resistance was operationalized as the time to task failure, which was defined as the inability to sustain contraction force within 5% of the 30% MVC target for more than 3 s. Participants received visual and auditory feedback to maintain the target exercise intensity throughout the session, and strong verbal encouragement was provided consistently throughout the fatiguing bout.

### 2.4 Cardiovascular function measurements

#### 2.4.1 Flow‐mediated dilation

Right‐brachial artery FMD was assessed using ultrasound (Arietta V750, Fujifilm, Tokyo, Japan) with a 7.5‐MHz linear array probe and 5‐MHz Doppler, positioned ~4 cm above the antecubital fossa and secured with a stereotactic clamp per standard guidelines (Corretti et al., 2002; Thijssen et al., 2019). Reactive hyperemia was induced by rapid cuff deflation following a forearm occlusion of 5‐min at 250 mmHg. Intraluminal diameter changes were tracked with automated edge‐detection software (FMD studio, Quipu srl, Pisa, Italy), allowing precise measurement (Thijssen et al., 2019). Doppler measurements of blood velocity with an insonation angle of 60° allowed the calculation of FMD main stimuli‐shear rate as: 4 × (peak blood velocity/*D*_bas_) (Thijssen et al., 2019). Shear rate area under the curve to peak dilation (SR AUC *D*_peak_) was calculated as a time integral from cuff release to peak brachial artery diameter. All FMD analyses were conducted offline using the FMD studio. FMD is presented as a relative diameter change (%FMD = (*D*_peak_ −*D*_bas_/*D*_bas_) × 100). Given the non‐linear ratio between peak diameter (*D*_peak_) and baseline diameter (*D*_bas_), %FMD was allometrically scaled for *D*_bas_ (Marôco et al., 2022, 2023). All FMD scans were conducted by the same researcher (CV of 9.9%).

#### 2.4.2 Brachial blood flow

Brachial blood flow ($\dot{Q}$) was estimated using the following equation: $\dot{Q}$ (mL/min) = [blood velocity × *π* × (vessel diameter/2)^2^ × 60], using a tailored R script. FMD Studio raw files, with 1‐s smooth averages for brachial artery diameter and blood velocity, were imported into R. Brachial flow was estimated over specific time windows using a 95% trimmed mean to reduce the influence of outliers. Mean blood flow was calculated over the 30‐s preceding exercise (baseline), the final 30‐s of the IHG, and at several time points following exercise cessation (i.e., the first 5, 10, and 15‐s, as well as the final 15‐s of the 3‐min recovery period). To normalize for pressor responses to IHG, brachial conductance was calculated as follows: $\dot{Q}$/MAP. In addition, sustained FMD after IHG was calculated as described for traditional FMD, but using peak diameters and shear rates in the first 3‐min after isometric handgrip exercise cessation. All brachial flow analyses during IHG were conducted by the same researcher (inter‐day CV of 12.94%).

#### 2.4.3 Blood pressure and heart rate

Right‐arm brachial BP was measured three times at 1‐min intervals before and after IHG using an automated blood pressure cuff following 15‐min rest period (HEM‐7311‐ZSA; Omron Healthcare Co, Ltd., Kyoto, Japan) (Muntner et al., 2019). Measurements were accepted if they did not differ >4 mmHg, with the average of the last two used for data analysis (Muntner et al., 2019). Mean arterial pressure was estimated as: 2/3 DBP + 1/3 SBP. Subsequently, continuous beat‐to‐beat BP was recorded using a finger photoplethysmography system (Finapres Medical System, Amsterdam, The Netherlands), calibrated to reflect brachial pressures and interfaced with a PowerLab data acquisition system. Heart rate (HR) was derived from a single‐lead ECG, also recorded via PowerLab. Stroke volume (SV) was estimated from Model flow, which allow calculation of CO as HR × SV and total peripheral resistance (TPR) as MAP/CO. Both BP and HR were averaged over defined time windows: the 30‐s preceding exercise (baseline), the final 30‐s of the IHG exercise, and the same post‐exercise time points used for brachial blood flow (i.e., 5, 10, 15‐s, and the last 30‐s of the 3‐min recovery period).

### 2.5 Statistical analyses

Based on the significant sex‐by‐time interaction reported by Thompson et al. (2007) for brachial blood flow during isometric handgrip at 30% MVC, we estimated a partial Cohen's *f* of 0.25 from their absolute data. Given that an initial power analysis using this effect size suggested a sample of approximately half used by Thompson et al (*n* = 18 males, *n* = 20 females), we assumed a conservative standardized medium effect size of 0.20. The final a priori power analysis (G‐Power Version 3.1.9.3) indicated that a total of 28 participants would be sufficient to detect significant differences between sexes and time points (*α* = 0.05, 1 − *β* = 0.80). All statistical analyses were conducted using R (version 4.5.1), with an α of 0.05. The data are presented as mean (SD) unless otherwise stated. Differences in clinical and isometric handgrip characteristics across sex were tested using Welch's independent‐sample *t*‐tests.

Brachial flow and pressor responses to IHG were examined using linear mixed models fitted with restricted maximum likelihood (lmerTest package). Degrees of freedom for the *F*‐test were approximated via Satterthwaite's methods. Linear mixed models consisted of two fixed effects (Time, Sex), an interaction term, and a random intercept for each participant. Model residuals were assessed for normality using Q–Q plot inspection and the Shapiro–Wilk test, and for homogeneity using the Breusch–Pagan test. IHG‐induced changes in hemodynamic outcomes were controlled for pre‐exercise BP values, MVC, and arm circumference, entered separately and altogether into the models. Partial omega squares (*ω* ^2^) were calculated for all fixed effects and interactions and interpreted following benchmarks (small <0.05, medium <0.25, and large >0.25). *Post‐hoc* comparisons were conducted using Bonferroni correction for significant fixed effects and interactions. Cohen's dz effect sizes were computed for *post‐hoc* comparisons of interest and interpreted following Cohen's benchmarks (small: 0.20–0.49, medium: 0.50–0.79; large:≥0.80). Pearson correlation coefficients were computed to test the association between IHG‐induced changes in brachial flow and MAP with time‐to‐task failure.

## 3 RESULTS

### 3.1 Characteristics of the participants and fatiguing isometric exercise

Table 1 presents the characteristics of the participants and the isometric handgrip exercise. Young females compared to their male counterparts exhibited a smaller arm circumference (Δ = −3.30 cm, 95% CI: −5.87 to −0.88 cm, *p* = 0.012). Lower bSBP (Δ = −8 mmHg, 95% CI: −14 to −2 mmHg, *p* = 0.016) but not bDBP was observed in females. Resting HR was higher in young females than in males. Young females, compared to males, exhibited a lower 30%MVC (Δ = −4.28 kg, 95% CI: −10.36 to −6.09 kg, *p* < 0.001) but a greater time‐to‐task failure (Δ = 62 s, 95% CI: 12–113 s, *p* = 0.001).

**TABLE 1:** Characteristics of the participants and the fatiguing isometric handgrip exercise.

	Female, *n* = 15	Male, *n* = 14	*p*‐value
Age, years	24 (3)	23 (4)	0.471
Height, cm	164 (8)	176 (9)	0.001
Weight, kg	61.66 (11.66)	77.44 (17.93)	0.011
Body mass index, kg m^−2^	22.73 (3.18)	24.89 (5.25)	0.198
Waist circumference, cm	77.30 (11.28)	84.14 (15.03)	0.181
Arm circumference, cm	26.55 (3.36)	29.87 (3.33)	0.012
Fat mass, %	29.88 (7.17)	17.25 (5.73)	<0.001
bSBP, mmHg	101 (9)	108 (7)	0.016
bDBP, mmHg	70 (7)	65 (7)	0.086
FMD %	7.69 (2.86)	5.47 (2.45)	0.018
FMD sc, %	6.99 (2.14)	6.08 (2.39)	0.326
D_bas_, mm	2.96 (0.44)	3.82 (0.49)	<0.001
Dpeak, mm	3.18 (0.48)	4.02 (0.49)	<0.001
SR AUC D_peak_, s^−1^ × 10^3^	3.8 (1.7)	2.2 (1.3)	0.011
HR, bpm	66 (9)	56 (9)	0.005
Fatiguing isometric handgrip			
MVC, kg	20.31 (6.23)	34.56 (11.51)	0.002
30% MVC, kg	6.09 (1.87)	10.37 (3.45)	0.002
Time‐to fatigue, s	243 (80)	181 (50)	0.023
IHG‐FMD, %	13.63 (4.30)	12.52 (5.59)	0.553
IHG‐FMD sc, %	12.51 (4.04)	13.68 (5.24)	0.520
SR AUC total s^−1^ × 10^3^	23.3 (13.7)	15.3 (8.0)	0.009

*Note*: Data presented as mean (SD).

Abbreviations: bDBP, brachial diastolic blood pressure; bSBP, brachial systolic blood pressure; Dbas, brachial baseline diameter; Dpeak, peak brachial diameter; FMD, flow‐mediated dilation; HR, heart rate; IHG‐FMD, post‐isometric handgrip‐induced flow‐mediated dilation; MVC, maximum voluntary contraction; sc, scaled to baseline diameter; SR AUC *D*_peak_, shear rate area under the curve until peak diameter; SR AUC total, total shear rate area under the curve.

### 3.2 Brachial blood flow and hemodynamic response to fatiguing isometric handgrip exercise

**FIGURE 1:**
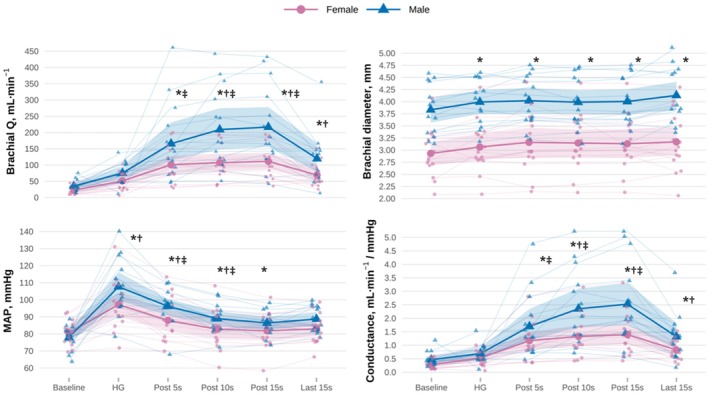
Brachial blood flow (Q), diameter, mean arterial pressure (MAP), and conductance responses to fatiguing isometric handgrip exercise in baseline (BAS) and control (CON) visits. *Different from baseline (*p* < 0.05); † Different from female (*p* < 0.05); ‡Different from handgrip (HG) (*p* < 0.05). Shaded ribbons corresponds to 95% confidence intervals.

Sex‐by‐time interactions were observed for brachial flow [*F* (5, 135) = 5.495, *p* < 0.001, ω^2^ = 0.14, Figure 1], SBP [*F* (5, 135) = 2.609, *p* = 0.0275, ω^2^ = 0.05], DBP [*F* (5, 135) = 2.573, *p* = 0.029, ω^2^ = 0.06], MAP (Table 2), and conductance (Table 3). Young females showed a preserved brachial blood flow during fatiguing IHG (Δ = 29 mL min^−1^s, 95% CI: −78 to 21 mL min^−1^s, *p* = 0.985, Cohen's dz. = 1.48 Figure 1), similar to those of males (Δ = 32 mL min^−1^, 95% CI: −85 to 21 mL min^−1^, *p* = 0.975, Cohen's dz. = 1.00). A smaller mean pressor response during IHG was observed in females (Δ = 17 mmHg, 95% CI: 10–23 mmHg, *p* < 0.001, Cohen's dz. = 1.47) when compared to males (Δ = 30 mmHg, 95% CI: 24–37 mmHg, *p* < 0.001, Cohen's dz. = 1.57). The 15‐s post‐exercise hyperemia was smaller in young females (Δ_15‐rest_ = 89 mL min^−1^, 95% CI: 39–138 mL min^−1^, *p* < 0.001, Cohen's dz. = 1.98) than in males (Δ_15‐rest_ = 182 mL min^−1^, 95% CI: 130–233 mL.min^−1^, *p* < 0.001, Cohen's dz. = 1.82). IHG‐FMD was similar across sexes (Δ_female‐male_ = 0.89%, 95% −2.11 to 3.89%, *p* = 0.553, Table 1). Both SBP and DBP returned to baseline within 10‐s post‐fatiguing IHG in young females but not in males (Table 2). Controlling for MAP did not change sex differences in post‐exercise hyperemia; consistent with the conductance response (Table 3). Percent change analyses (i.e., relative to baseline) aligned with the results of absolute analyses described (Table S1). Additional control for sex differences in arm circumference, brachial D, bMAP, and MVC did not affect brachial flow responses to IHG across sex (Table S2).

**TABLE 2:** Hemodynamic response to fatiguing isometric exercise.

	Pre	HG	Post 5 s	Post 10s	Post 15 s	Last 15 s	Time	Sex	Interaction
							*p* (*ω* ^2^)	*p* (*ω* ^2^)	*p* (*ω* ^2^)
HR, bpm									
Female	71 (10)	84 (12)^a^	75 (10)^a^, ^c^	72 (12)^a^, ^c^	70 (9)	69 (9)	**<0.001 (0.41)**	**0.001 (0.28)**	0.928 (0.00)
Male	57 (9)	75 (15)^a^	65 (10)^a^, ^c^	61 (9)^a^, ^c^	59 (12)	54 (18)			
SV, mL									
Female	49 (11)	49 (19)	51 (18)	53 (18)^a^, ^c^	56 (15)^a^, ^c^	53 (12)^a^, ^c^	**0.002 (0.09)**	**<0.001 (0.41)**	0.385 (0.00)
Male	74 (24)	82 (34)	89 (28)	90 (32)^a^, ^c^	94 (30)^a^, ^c^	84 (24)^a^, ^c^			
CO, L min^−1^									
Female	3.5 (1.0)	4.2 (1.9)^a^	3.8 (1.4)^a^	3.8 (1.5)^a^	4.0 (1.3)^a^	3.6 (1.1)	**<0.001 (0.15)**	**0.006 (0.21)**	0.187 (0.00)
Male	4.2 (1.2)	6.1 (3.1)^a^	5.7 (1.7)^a^	5.4 (1.4)^a^	4.5 (1.7)^a^	4.4 (0.7)			
TPR, mmHg L min^−1^									
Female	24.5 (5.4)	27.3 (11.2)	25.6 (8.7)	24.3 (8.1)	22.1 (5.7)	24.4 (6.0)	0.069 (0.04)	**0.041 (0.11**)	0.846 (0.00)
Male	21.0 (9.8)	21.3 (10.7)	19.5 (9.4)	19.2 (10.0)	18.1 (7.9)	19.7 (6.6)			
MAP, mmHg									
Female	81 (7)	97 (15)^a^	88 (12)^a^, ^c^	82 (10)	83 (9)	81 (6)	**<0.001 (0.64)**	0.114 (0.05)	**0.002 (0.10)**
Male	78 (7)	108 (16)^a^, ^b^	96 (10)^a^, ^b^, ^c^	89 (8)^a^, ^b^, ^c^	87 (7)^a^, ^b^, ^c^	86 (7)			
SBP, mmHg									
Female	101 (9)	118 (19)^a^	111 (17)^a^	106 (16)^c^	105 (14)^c^	105 (13)^c^	**<0.001 (0.48)**	**0.003 (0.25)**	**0.028 (0.05)**
Male	108 (7)	141 (18)^a^, ^b^	132 (22)^a^, ^b^, ^c^	126 (19)^a^, ^b^, ^c^	120 (17)^a^, ^b^, ^c^	113 (18)^c^			
DBP, mmHg									
Female	70 (7)	83 (12)^a^	74 (10)^a^, ^c^	70 (10)	69 (9)	70 (7)	**<0.001 (0.54)**	0.500 (0.00)	**0.029 (0.05)**
Male	65 (7)	90 (18)^a^, ^b^	79 (10)^a^, ^b^, ^c^	72 (8)	70 (7)	73 (9)			
PP, mmHg									
Female	31 (8)	35 (12)^a^	37 (11)^a^	36 (10)^a^	36 (9)^a^	35 (11)	**0.008 (0.08)**	**0.001 (0.30)**	0.723 (0.00)
Male	43 (6)	52 (13)^a^	53 (20)^a^	53 (19)^a^	50 (11)^a^	46 (16)			

*Note*: Data presented as mean (SD).

Abbreviations: CO, cardiac output; DBP, diastolic blood pressure; HR, heart rate; MAP, mean arterial pressure; PP, pulse pressure; SBP, systolic blood pressure; SV, stroke volume; TPR, total peripheral resistance.

^a^ Different from baseline (*p* < 0.05).

^b^ Different from female (*p* < 0.05).

^c^ Different from handgrip (*p* < 0.05).

**TABLE 3:** Brachial blood flow response to fatiguing isometric exercise.

	Pre	HG	Post 5 s	Post 10s	Post 15 s	Last 15 s	Time	Sex	Interaction
							*p* (*ω* ^2^)	*p* (*ω* ^2^)	*p* (*ω* ^2^)
Q, mL min^−1^									
Female	22 (13)	51 (28)	101 (55)^a^, ^c^	108 (54)^a^, ^c^	111 (55)^a^, ^c^	69 (36)^a^, ^d^	**<0.001 (0.59)**	**0.014 (0.17)**	**<0.001 (0.14)**
Male	35 (18)	75 (36)	166 (120)^a^, ^c^	209 (122)^a^, ^b^, ^c^	217 (126)^a^, ^b^, ^c^	120 (86)^a^, ^d^			
D, mm									
Female	2.93 (0.49)	3.06 (0.52)	3.16 (0.61)^a^, ^c^	3.15 (0.58)^a^, ^c^	3.13 (0.56)^a^, ^c^	3.17 (0.61)^a^, ^c^	**<0.001 (0.27)**	**<0.001 (0.40)**	0.635 (0.00)
Male	3.83 (0.52)	3.99 (0.48)	4.02 (0.52)^a^, ^c^	3.99 (0.48)^a^, ^c^	4.00 (0.53)^a^, ^c^	4.13 (0.57)^a^, ^c^			
BV, cm s^−1^									
Female	5.30 (2.09)	11.45 (5.65)^a^	20.49 (7.53)^a^, ^c^	22.25 (7.80)^a^, ^c^	23.11 (7.23)^a^, ^c^	15.14 (7.29)^a^, ^d^	**<0.001 (0.69)**	0.780 (0.00)	0.138 (0.02)
Male	4.91 (1.68)	10.04 (4.07)^a^	20.27 (11.74)^a^, ^c^	26.24 (11.75)^a^, ^c^	26.98 (12.37)^a^, ^c^	13.92 (6.56)^a^, ^d^			
SR, s^−1^									
Female	37 (15)	77 (43)^a^	135 (61)^a^, ^c^	148 (64)^a^, ^c^	150 (61)^a^, ^c^	106 (62)^a^, ^d^	**<0.001 (0.68)**	0.108 (0.06)	0.534 (0.00)
Male	26 (9)	51 (21)^a^	100 (55)^a^, ^c^	130 (54)^a^, ^c^	133 (56)^a^, ^c^	67 (29)^a^, ^d^			
Q/MAP									
Female	0.28 (0.16)	0.53 (0.29)	1.17 (0.67)^a^, ^c^	1.33 (0.74)^a^, ^c^	1.40 (0.78)^a^, ^c^	0.83 (0.37)^a^, ^d^	**<0.001 (0.60)**	**0.032 (0.18)**	**<0.001 (0.11)**
Male	0.47 (0.28)	0.69 (0.36)	1.70 (1.20)^a^, ^b^, ^c^	2.36 (1.41)^a^, ^b^, ^c^	2.52 (1.54)^a^, ^b^, ^c^	1.32 (0.90)^b^, ^c^, ^d^			

*Note*: Data presented as mean (SD).

Abbreviations: BV, blood velocity; D, diameter; Q, blood flow; SR, shear rate.

^a^ Different from baseline (*p* < 0.05).

^b^ Different from female (*p* < 0.05).

^c^ Different from handgrip (*p* < 0.05).

^d^ Different from post15s.

Time effects were noted for HR, SV, and CO (Table 2). In both sexes, CO increments (Δ = 1.33 L min^−1^, 95% CI: 0.52–2.13 L min^−1^, *p* < 0.001, Cohen's dz. = 0.49) during fatiguing IHG were driven by HR (Δ = 15 b min^−1^, 95% CI: 9–20 b min^−1^, *p* < 0.001, Cohen's dz. = 0.74), as SV remained unaltered. Conversely, persistent elevations in CO during 15‐s post IHG were driven by SV.

### 3.3 Association of brachial blood flow and pressure response with time‐to‐task‐failure

Neither changes during the IHG of the brachial flow [Female: *r* (28) = 0.314, *p* = 0.098; Male: *r* (25) = 0.144, *p* = 0.323] nor MAP [Female: *r* (28) = 0.012, *p* = 0.925; Male: *r* (25) = 0.144, *p* = 0.472] were associated with time‐to‐task failure. MVC was not associated with time‐to‐task failure in females [*r* (28) = 0.1787, *p* = 0.347], and males [*r* (25) = −0.195, *p* = 0.333].

## 4 DISCUSSION

The main findings of this investigation were that: (1) time‐to‐task failure was longer in females than in males; (2) brachial blood flow was similarly preserved during IHG across sex, despite a greater male pressor response; (3) males compared to females exhibited both a greater post‐exercise hyperemia and an elevated BP; and (4) sustained FMD was similar across sex. Collectively, the greater fatigue resistance of females compared to males during IHG appears unrelated to brachial flow and pressor responses, refuting our hypotheses. Future research should clarify mechanisms behind sex differences in IHG fatigability, which may limit use as an antihypertensive exercise modality, and assess whether greater post‐exercise hyperemia in males benefits blood pressure control.

### 4.1 Brachial blood flow and pressure response during fatiguing isometric handgrip exercise

Although the greater ability of females to preserve blood flow is consistently advanced as a mechanism for sex differences in isometric exercise fatigability, the limited studies with rigorous measurements of brachial flow dispute this contention. Only Thompson et al. (2007) and we have examined ultrasound‐derived brachial blood flow during fatiguing IHG across sexes, rather than indirect methodologies or complete vessel occlusion. In contrast to Thompson et al. (2007), who reported similar increases in brachial flow at 20% MVC in both sexes, we observed no change at 30% MVC. Despite using a higher intensity (30% vs. 20% MVC), it is unlikely to explain the conflicting findings, as Thompson et al. (2007) also reported sex‐independent increases at 50% MVC. In turn, methodological heterogeneity is a likely candidate behind the discrepancy. While we acquired diameters and blood velocity simultaneously, Thompson et al. obtained these on different days using resting diameters to estimate brachial blood flow, which masks exercise‐induced variability and rapid dynamic changes in flow due to diameter changes. In accordance with Poiseuille's equation, the small non‐significant increase in brachial diameter during IHG preserved brachial flow across sexes, suggesting minimal, if any, arterial compression and a limited influence of flow responses on sex differences in low‐intensity isometric exercise fatigability.

Considering Poiseuille's postulate of perfusion pressure as a key driver of flow, we examined beat‐to‐beat BP during IHG to elucidate the uncertain role of pressor responses for sex differences observed in fatiguability (Hunter & Enoka, 2001; Keller et al., 2022). While the small pressor response of females during exercise is expected to reduce brachial flow and is thought to contribute greater fatigue resistance than that of males, our findings refute such observations. Initially surprising, especially after normalizing for the nearly twofold smaller rise in mean pressure among females, the similar brachial conductance between sexes during IHG underscores the dominant role of arterial diameter in governing blood flow (i.e., radius fourth power). Moreover, the sex‐specific BP response observed was not associated with time‐to‐task failure, aligning with recent evidence indicating that the small pressor response of females does not induce greater fatigue‐resistance to IHG compared to males. Taken together, brachial flow and pressure appear not to drive sex differences in IHG fatigability.

The mechanisms behind the smaller exercise pressor response of young females during IHG remain unclear, but are mainly attributed to an attenuated firing of the exercise pressor reflex (Hogarth et al., 2006; Hunter & Enoka, 2001; Jarvis et al., 2011; Smith et al., 2019). Reflexively‐mediated increases in BP during intense or fatiguing exercise arise primarily from sympathetic‐driven elevations in total peripheral resistance (Fadel, 2008; Grotle et al., 2023). Given reports of higher muscle sympathetic activity in males (D'souza et al., 2023; Jarvis et al., 2011) and greater β‐adrenergic–mediated vasodilation in females (Jacob et al., 2025; Kneale et al., 2000), smaller TPR and vasoconstrictor responses would be expected in females. However, TPR remained unchanged, and brachial diameter increased non‐significantly during IHG, with no sex differences observed. These findings suggest that locally‐mediated functional sympatholysis via vasodilators (e.g., ATP) is comparable between sexes, in line with recent evidence (Saltin & Mortensen, 2012; Teixeira et al., 2022). Thus, it follows, as per Ohm's law, that the observed pressor response resulted mainly from an HR‐mediated rise in CO. Although females exhibited lower absolute CO, the smaller pressor response is not fully explained by CO, as increases during IHG were similar across sex. Alternatively, the lower absolute exercise workload in females is suggested to contribute to their smaller pressor responses via reduced mechanoafferent firing of the exercise pressor reflex compared with males (Ives et al., 2013; Lee et al., 2021). Indeed, sex differences in the exercise pressor reflex appear to originate at the afferent level, specifically at the mechanically (group III) sensitive fibers, and not at cardiovascular control centers (Ives et al., 2013; Jarvis et al., 2011; Keller et al., 2022). Still, when pressor responses were covaried for MVC, sex differences persisted.

### 4.2 Brachial blood flow and pressure response after fatiguing isometric handgrip exercise

We extend that the greater post‐exercise hyperemia in males compared to females is unrelated to sex dimorphism in IHG fatigability (Hunter et al., 2006, 2009). The greater post‐exercise hyperemia in males is consistently interpreted as evidence that females show a greater muscle perfusion during IHG contraction. Even if muscle‐specific assessments of perfusion (e.g., MRI‐arterial spin labeling) remain unexplored, Thompson et al. and our results show no sex dimorphism in Doppler‐derived brachial flow response during low‐intensity IHG. Of note, these observations do not preclude that IHG induces a greater ischemic stimulus in males, only that it is unrelated to brachial flow and BP. Despite BP remaining elevated in the initial 10‐s post only in males, it failed to explain the sex difference in post‐exercise hyperemia, as conductance mirrored blood flow results.

Sex dimorphism in energy metabolism plausibly contributes to sex differences in isometric exercise fatigability and post‐exercise hyperemia (Hunter, 2014, 2016). The greater reliance of males on glycolysis during isometric exercise, reflecting their larger cross‐sectional area of fatigable type II muscle fibers compared to females, is considered a key mechanism behind sex differences in fatiguability (Hunter, 2014, 2016; Russ et al., 2003). Although not universally supported (Laginestra et al., 2025; Russ et al., 2005), the greater hydrogen ion release from glycolytic reactions, not lactate production as once thought (which can consume hydrogen ions) (Robergs et al., 2018), may affect muscle excitation‐contraction coupling in males, a process associated with fatigue (Cairns & Lindinger, 2025; Hunter, 2014, 2016). Interestingly, recent evidence suggests that inorganic phosphorus metabolites, rather than hydrogen ions, are more consistently associated with muscle fatigue, but intramuscular accumulation of these metabolites was similar between sexes (Laginestra et al., 2025; Lewis et al., 2025). Thus, it appears that the in vivo metabolic basis of muscle fatigue is similar between sexes (Laginestra et al., 2025). In fact, lactate, once considered a dead‐end fatiguing metabolite, is now viewed as a major metabolic intermediate with actions on substrate utilization, cell signaling, and vasomotor tonus (Brooks et al., 2022). Lactate induces flow‐independent vasorelaxation of smooth muscle cells (Hein et al., 2006; Homilius et al., 2025; Mori et al., 1998), which may offset the greater compressional effects from higher absolute workloads in males and explain the absence of vasoconstriction during IHG. Still, MVC was not associated with post‐exercise brachial blood flow in both sexes. Moreover, the similar increase in diameter across sexes leaves the basis of sex dimorphism in post‐exercise hyperemia uncertain. Alternatively, the greater post‐exercise hyperemia in males may reflect an overshoot response to the greater desaturation observed during isometric handgrip exercise in comparison to females, with reductions in oxygenation independent of total blood volume and intramuscular pressure (Keller et al., 2022). These sex‐specific oxygenation patterns are mechanistically linked to mitochondrial respiration, with males exhibiting a smaller oxidative capacity and efficiency, along with a pronounced dependence on Complex II activity (Cardinale et al., 2018; Giuriato et al., 2025). However, it remains uncertain whether these sex differences in oxygenation and mitochondrial respiration account for the greater fatigue resistance noted in females during isometric exercise (Keller et al., 2022).

Although our males exhibited greater post‐exercise hyperemia, sustained stimulus FMD was identical to that of young females following IHG cessation, contrasting with the observed and well‐documented sex difference in traditional FMD (Celermajer et al., 1994; Holder et al., 2021). Curiously, traditional FMD was lower than sustained FMD, aligning with limited reports of differential responses to stressors (i.e., high‐fat meals, mental stress) (Padilla et al., 2006; Szijgyarto et al., 2013). Collectively, these findings suggest that the endothelium distinguishes between transient and sustained shear stress, the key stimulus for FMD, via distinct vasodilatory mechanisms, possibly underlying sex dimorphism. The higher transient shear‐stress mediated vasodilation in young females is typically attributed to estrogen regulatory actions regarding calcium‐dependent or Akt activation of endothelial nitric oxide synthase (Goetz et al., 1999; Florian et al., 2004). Conversely, sustained shear‐stress‐mediated dilation appears more dependent on the balance between vasodilatory and vasoconstrictive factors (endothelin‐1) than on calcium and nitric oxide, with potential sex differences unclear (Tremblay & Pyke, 2018). Methodologically, the causal relationship between shear stress (indexed by shear rate) and flow‐mediated responses is confounded by inverse associations with baseline diameter (Atkinson & Batterham, 2013). Indeed, scaling for *D*_bas_ abolished the greater traditional FMD of females, rendering unclear whether FMD responses should be normalized to SR (Atkinson, 2014). Despite greater shear stimuli and smaller diameters, females showed sustained FMD comparable to males, suggesting that post‐IHG dilation, unlike dynamic handgrip, may be flow‐independent, particularly given the greater male hyperemia. Future work should clarify the mechanisms and relevance of sustained FMD across dynamic and isometric exercise paradigms.

### 4.3 Limitations

Brachial blood flow responses were not expressed in relative terms, challenging interpretation given sex differences in muscle mass and strength. However, covariate control for MVC and arm circumference unchanged absolute responses, consistent with reports of similar absolute and relative responses to lower‐intensity IHG (Thompson et al., 2007). Although we conducted an a priori power analysis, our inferences regarding brachial blood flow during isometric handgrip exercise are likely underpowered. In fact, we observed a non‐significant increase across sexes despite a large effect size noted (Cohen's dz. >0.8), suggesting limited power to detect relevant changes. Comparing brachial flow and pressor responses at the same absolute IHG intensity robustly controls for sex difference in strength; still, this approach reduces ecological validity. Furthermore, without quantification of muscle sympathetic nerve activity, post‐exercise ischemia (exercise pressor reflex), or circulating vasoactive (e.g., nitric oxide, endothelin) and metabolic (e.g., lactate), mechanistic interpretations of brachial flow and pressor responses, along with contributions to isometric fatigability, are limited. Additionaly, we did not collect biomarkers of fatigue to confirm upper limb muscle fatigue. However, there is current lack of consensus in which specific fatigue biomarker to prioritize, given the substantial inter‐individual variability and limited specificity of both objective (e.g., hydrogen ions, reactive oxygen species) and subjective biomarkers (rate of fatigue) (Finsterer, 2012; Micklewright et al., 2017; Wan et al., 2017). Since cardiovascular testing did not account for the menstrual cycle, we cannot exclude the effects of hormonal fluctuations on brachial flow and pressor responses or fatiguability. However, growing evidence shows little to no variation in BP and vascular function (FMD) at rest or during exercise across menstrual or contraceptive cycles (Williams et al., 2020; D'Urzo et al., 2018; Hartwich et al., 2013; Kim et al., 2012; Priest et al., 2018; Shenouda et al., 2018; Yu et al., 2014). Lastly, our results only apply to young males and females, and whether brachial flow and pressor responses remain unlinked with IHG fatiguability in older and or clinical populations is unknown.

## 5 CONCLUSIONS

Brachial flow and pressor responses appear unrelated to the greater fatigue resistance of young females compared to males. The greater post‐exercise hyperemia in males suggests a greater ischemic stimulus, which is not directly related to blood flow or to a greater sustained FMD compared to females. Future research should determine the mechanisms behind sex differences in IHG fatigability and whether sustained FMD after IHG is flow‐independent.

## AUTHOR CONTRIBUTIONS

**João L. Marôco:** Conceptualization; data curation; formal analysis; investigation; methodology; visualization. **Joseph Scangas:** Data curation; investigation. **Megan Borges:** Data curation; investigation. **Eric Bracken:** Data curation; investigation. **Anton Pecha:** Data curation; investigation. **Tracy Baynard:** Conceptualization; project administration; supervision. **Bo Fernhall:** Conceptualization; project administration; supervision; validation.

## FUNDING INFORMATION

No funding was received to support this study.

## CONFLICT OF INTEREST STATEMENT

The authors declare no conflicts of interest.

## ETHICS STATEMENT

The study was approved by the IRB of the University of Massachusetts Boston and conformed to the Declaration of Helsinki (2024 revision). All participants provided written informed consent.

## Supporting information


**Table S1.** Brachial blood flow and mean pressure responses during and a 15‐s post‐fatiguing isometric handgrip presented as relative change to baseline.

**Table S2.** Linear mixed models for brachial blood flow, mean arterial pressure (MAP), and systolic blood pressure (SBP) controlled for brachial diameter, arm circumference, and maximum voluntary contraction (MVC).

## Data Availability

The data that support the findings of this study are available from the corresponding author, Bo Fernhall, upon reasonable request.

## References

[phy270871-bib-0001] Alves, A. J. , Wu, Y. , Lopes, S. , Ribeiro, F. , & Pescatello, L. S. (2022). Exercise to treat hypertension: Late breaking news on exercise prescriptions that FITT. Current Sports Medicine Reports, 21, 280–288. www.acsm‐csmr.org 35946847 10.1249/JSR.0000000000000983

[phy270871-bib-0002] Atkinson, G. (2014). Shear rate normalisation is not essential for removing the dependency of flow‐ mediated dilation on baseline artery diameter: Past research revisited. Physiological Measurement, 35, 1–14. 25139144 10.1088/0967-3334/35/9/1825

[phy270871-bib-0003] Atkinson, G. , & Batterham, A. M. (2013). The percentage flow‐mediated dilation index: A large‐sample investigation of its appropriateness, potential for bias and causal nexus in vascular medicine. Vascular Medicine (United Kingdom), 18, 354–365. 10.1177/1358863X13508446 24172228

[phy270871-bib-0004] Barbosa, T. C. , Vianna, L. C. , Fernandes, I. A. , Prodel, E. , Rocha, H. N. M. , Garcia, V. P. , Rocha, N. G. , Secher, N. H. , & Nobrega, A. C. L. (2016). Intrathecal fentanyl abolishes the exaggerated blood pressure response to cycling in hypertensive men. Journal of Physiology, 594, 715–725. 10.1113/JP271335 26659384 PMC5341703

[phy270871-bib-0005] Barnes, W. S. (1980). The relationship between maximum isometric strength and intramuscular circulatory occlusion. Ergonomics, 23, 351–357. 10.1080/00140138008924748 7202390

[phy270871-bib-0006] Brooks, G. A. , Arevalo, J. A. , Osmond, A. D. , Leija, R. G. , Curl, C. C. , & Tovar, A. P. (2022). Lactate in contemporary biology: A phoenix risen. Journal of Physiology, 600, 1229–1251. 10.1113/JP280955 33566386 PMC9188361

[phy270871-bib-0007] Cairns, S. P. , & Lindinger, M. I. (2025). Lactic acidosis: Implications for human exercise performance. European Journal of Applied Physiology, 125, 1761–1795. 40088272 10.1007/s00421-025-05750-0 PMC12227488

[phy270871-bib-0008] Cardinale, D. A. , Larsen, F. J. , Schiffer, T. A. , Morales‐Alamo, D. , Ekblom, B. , Calbet, J. A. L. , Holmberg, H. C. , & Boushel, R. (2018). Superior intrinsic mitochondrial respiration in women than in men. Frontiers in Physiology, 9, 1133. 10.3389/fphys.2018.01133 30174617 PMC6108574

[phy270871-bib-0009] Celermajer, D. , Sorensen, K. , Spiegelhalter, D. , Georgakopoulos, D. , Robinson, J. , & Deanfield, J. (1994). Aging is associated with endothelial dysfunction in healthy men years before the age‐related decline in women. Journal of the American College of Cardiology, 24, 471–476. 10.1016/0735-1097(94)90305-0 8034885

[phy270871-bib-0010] Chuah, S. S. , Woolfson, P. I. , Pullan, B. R. , & Lewis, P. S. (2004). Plethysmography without venous occlusion for measuring forearm blood flow: Comparison with venous occlusive method. Clinical Physiology and Functional Imaging, 24, 296–303. 10.1111/j.1475-097X.2004.00566.x 15383087

[phy270871-bib-0011] Corretti, M. C. , Anderson, T. J. , Benjamin, E. J. , Celermajer, D. , Charbonneau, F. , Creager, M. A. , Deanfield, J. , Drexler, H. , Gerhard‐Herman, M. , Herrington, D. , Vallance, P. , Vita, J. , & Vogel, R. (2002). Guidelines for the ultrasound assessment of endothelial‐dependent flow‐mediated vasodilation of the brachial artery. Journal of the American College of Cardiology, 39, 257–265. 10.1016/S0735-1097(01)01746-6 11788217

[phy270871-bib-0012] D'souza, A. W. , Takeda, R. , Manabe, K. , Hissen, S. L. , Washio, T. , Coombs, G. B. , Sanchez, B. , Fu, Q. , Shoemaker, J. K. , Fu, Q. , & Shoemaker, C.‐J.K. (2023). The interactive effects of age and sex on the neuro‐cardiovascular responses during fatiguing rhythmic handgrip exercise. The Journal of Physiology, 601, 2877–2898. 10.1113/JP284517#support-information-section 37083007

[phy270871-bib-0013] D'Urzo, K. , King, T. J. , Williams, J. S. , Silvester, M. D. , & Pyke, K. E. (2018). The impact of menstrual phase on brachial artery flow‐mediated dilatation during handgrip exercise in healthy premenopausal women. Experimental Physiology, 103, 291–302. 29083061 10.1113/EP086311

[phy270871-bib-0014] Fadel, P. J. (2008). Arterial baroreflex control of the peripheral vasculature in humans: Rest and exercise. Medicine and Science in Sports and Exercise, 40, 2055–2062. 10.1249/MSS.0b013e318180bc80 18981944

[phy270871-bib-0015] Finsterer, J. (2012). Biomarkers of peripheral muscle fatigue during exercise. BMC Musculoskeletal Disorders, 13, 218. 23136874 10.1186/1471-2474-13-218 PMC3534479

[phy270871-bib-0016] Florian, M. , Lu, Y. , Angle, M. , & Magder, S. (2004). Estrogen induced changes in Akt‐dependent activation of endothelial nitric oxide synthase and vasodilation. Steroids, 69, 637–645. 10.1016/j.steroids.2004.05.016 15465108

[phy270871-bib-0017] Giuriato, G. , Barbi, C. , Laginestra, F. G. , Andani, M. E. , Favaretto, T. , Martignon, C. , Pedrinolla, A. , Vernillo, G. , Moro, T. , Franchi, M. , Romanelli, M. G. , Schena, F. , & Venturelli, M. (2025). Mitochondrial influence on performance fatigability: Considering sex variability. Medicine and Science in Sports and Exercise, 57, 376–389. 10.1249/MSS.0000000000003558 39231737

[phy270871-bib-0018] Gliemann, L. , Mortensen, S. P. , & Hellsten, Y. (2018). Methods for the determination of skeletal muscle blood flow: Development, strengths and limitations. European Journal of Applied Physiology, 118, 1081–1094. 29756164 10.1007/s00421-018-3880-5

[phy270871-bib-0019] Goetz, R. M. , Prabhakar, P. , Cho, M. R. , Michel, T. , & Golan, D. E. (1999). Estradiol induces the calcium‐dependent translocation of endothelial nitric oxide synthase. Proceedings of the National Academy of Sciences of the United States of America, 96, 2788–2793. www.pnas.org 10077589 10.1073/pnas.96.6.2788 PMC15847

[phy270871-bib-0020] Greaney, J. L. , Edwards, D. G. , Fadel, P. J. , & Farquhar, W. B. (2015). Rapid onset pressor and sympathetic responses to static handgrip in older hypertensive adults. Journal of Human Hypertension, 29, 402–408. 10.1038/jhh.2014.106 25471615

[phy270871-bib-0021] Grotle, A. K. , Langlo, J. V. , Holsbrekken, E. , Stone, A. J. , Tanaka, H. , & Fadel, P. J. (2023). Age‐related alterations in the cardiovascular responses to acute exercise in males and females: Role of the exercise pressor reflex. Frontiers in Physiology, 14, 1287392. 38028783 10.3389/fphys.2023.1287392 PMC10652405

[phy270871-bib-0022] Hartwich, D. , Aldred, S. , & Fisher, J. P. (2013). Influence of menstrual cycle phase on muscle metaboreflex control of cardiac baroreflex sensitivity, heart rate and blood pressure in humans. Experimental Physiology, 98, 220–232. 10.1113/expphysiol.2012.066498 22613743

[phy270871-bib-0023] Hein, T. W. , Xu, W. , & Kuo, L. (2006). Dilation of retinal arterioles in response to lactate: Role of nitric oxide, guanylyl cyclase, and ATP‐sensitive potassium channels. Investigative Ophthalmology & Visual Science, 47, 693–699. 10.1167/iovs.05-1224 16431969

[phy270871-bib-0024] Hogarth, A. J. , Mackintosh, A. F. , & Mary, D. A. (2006). Gender‐related differences in the sympathetic vasoconstrictor drive of normal subjects. Clinical Science, 112, 353–361. 10.1042/CS20060288 17129210

[phy270871-bib-0025] Holder, S. M. , Bruno, R. M. , Shkredova, D. A. , Dawson, E. A. , Jones, H. , Hopkins, N. D. , Hopman, M. T. E. , Bailey, T. G. , Coombes, J. S. , Askew, C. D. , Naylor, L. , Maiorana, A. , Ghiadoni, L. , Thompson, A. , Green, D. J. , & Thijssen, D. H. J. (2021). Reference intervals for brachial artery flow‐mediated dilation and the relation with cardiovascular risk factors. Hypertension, 77, 1469–1480. 10.1161/hypertensionaha.120.15754 33745297

[phy270871-bib-0026] Homilius, C. , Seefeldt, J. M. , Hansen, J. , Nielsen, R. , de Paoli, F. V. , & Boedtkjer, E. (2025). Lactate orchestrates metabolic hemodynamic adaptations through a unique combination of venocontraction, artery relaxation, and positive inotropy. Acta Physiologica, 241, e70037. 10.1111/apha.70037 40167405 PMC11960580

[phy270871-bib-0027] Hunter, S. K. (2014). Sex differences in human fatigability: Mechanisms and insight to physiological responses. Acta Physiologica, 210, 768–789. 10.1111/apha.12234 24433272 PMC4111134

[phy270871-bib-0028] Hunter, S. K. (2016). The relevance of sex differences in performance fatigability. Medicine and Science in Sports and Exercise, 48, 2247–2256. 10.1249/MSS.0000000000000928 27015385 PMC5349856

[phy270871-bib-0029] Hunter, S. K. , & Enoka, R. M. (2001). Sex differences in the fatigability of arm musclesdepends on absolute force during isometric contractions. Journal of Applied Physiology, 91, 2686–2694. http://www.jap.org2686 11717235 10.1152/jappl.2001.91.6.2686

[phy270871-bib-0030] Hunter, S. K. , & Enoka, R. M. (2001). Sex differences in the fatigability of arm muscles depends on absolute force during isometric contractions. Journal of Applied Physiology, 91, 2686–2694. http://www.jap.org2686 11717235 10.1152/jappl.2001.91.6.2686

[phy270871-bib-0031] Hunter, S. K. , Griffith, E. E. , Schlachter, K. M. , & Kufahl, T. D. (2009). Sex differences in time to task failure and blood flow for an intermittent isometric fatiguing contraction. Muscle & Nerve, 39, 42–53. 10.1002/mus.21203 19086076

[phy270871-bib-0032] Hunter, S. K. , Schletty, J. M. , Schlachter, K. M. , Griffith, E. E. , Polichnowski, A. J. , Ng, A. V. , & Hunter, S. K. (2006). Active hyperemia and vascular conductance differ between men and women for an isometric fatiguing contraction. Journal of Applied Physiology (1985), 101, 140–150. 10.1152/japplphysiol.01567.2005.-To 16601303

[phy270871-bib-0033] Ives, S. J. , Mcdaniel, J. , Witman, M. A. H. , & Richardson, R. S. (2013). Passive limb movement: Evidence of mechanoreflex sex specificity. American Journal of Physiology. Heart and Circulatory Physiology, 304, 154–161. 10.1152/ajpheart.00532.2012.-Previous PMC3543682 23086995

[phy270871-bib-0034] Jacob, D. W. , Shariffi, B. , Bond, B. J. , Boyes, N. G. , Martin, S. A. , Gonsalves, A. M. , Harper, J. L. , Bostick, B. P. , & Limberg, J. K. (2025). b‐adrenergic receptors mediate sex differences in vasodilation but not sympathetic‐mediated vasoconstriction during hypoxia. American Journal of Physiology. Heart and Circulatory Physiology, 329, H233–H240. 10.1152/ajpheart.00192.2025 40465513 PMC12213120

[phy270871-bib-0035] Jarvis, S. S. , Vangundy, T. B. , Melyn Galbreath, M. , Shibata, S. , Okazaki, K. , Reelick, M. F. , Levine, B. D. , & Fu, Q. (2011). Sex differences in the modulation of vasomotor sympathetic outflow during static handgrip exercise in healthy young humans. American Journal of Physiology. Regulatory, Integrative and Comparative Physiology, 301, 193–200. 10.1152/ajpregu.00562.2010.-Sex PMC3129874 21508291

[phy270871-bib-0036] Keller, J. L. , Kennedy, K. G. , Hill, E. C. , Fleming, S. R. , Colquhoun, R. J. , & Schwarz, N. A. (2022). Handgrip exercise induces sex‐specific mean arterial pressure and oxygenation responses but similar performance fatigability. Clinical Physiology and Functional Imaging, 42, 127–138. 10.1111/cpf.12739 34979052

[phy270871-bib-0037] Kent‐Braun, J. A. , Ng, A. V. , Doyle, J. W. , Towse, T. F. , & Braun, K. (2002). Human skeletal muscle responses vary with age and gender during fatigue due to incremental isometric exercise. Journal of Applied Physiology (1985), 93, 1813–1823. 10.1152/japplphysiol.00091.2002.-The 12381770

[phy270871-bib-0038] Kim, A. , Deo, S. H. , Fisher, J. P. , & Fadel, P. J. (2012). Effect of sex and ovarian hormones on carotid baroreflex resetting and function during dynamic exercise in humans. Journal of Applied Physiology, 112, 1361–1371. 10.1152/japplphysiol.01308.2011 22267388 PMC3331588

[phy270871-bib-0039] Kneale, B. J. , Chowienczyk, P. J. , Brett, S. E. , Coltart, D. J. , & Ritter, J. M. (2000). Gender differences in sensitivity to adrenergic agonists of forearm resistance vasculature. Journal of the American College of Cardiology, 36, 1233–1238. 10.1016/S0735-1097(00)00849-4 11028476

[phy270871-bib-0040] Laginestra, F. G. , Broxterman, R. M. , Iannetta, D. , Lewis, M. T. , Kofoed, J. S. , Craig, J. C. , Stoddard, G. , Layec, G. , Jeong, E.‐K. , & Amann, M. (2025). The influence of biological sex on the metabolic basis of skeletal muscle fatigue in vivo. he Journal of Physiology, 603, 6881–6894. 10.1113/JP289709 PMC13570562 40961462

[phy270871-bib-0041] Lee, J. B. , Notay, K. , Seed, J. D. , Nardone, M. , Omazic, L. J. , & Millar, P. J. (2021). Sex differences in muscle Metaboreflex activation after static handgrip exercise. Medicine and Science in Sports and Exercise, 53, 2596–2604. 10.1249/MSS.0000000000002747 34310499

[phy270871-bib-0042] Lewis, M. T. , Laginestra, F. G. , Craig, J. C. , Amann, M. , Richardson, R. S. , Wiseman, R. W. , & Broxterman, R. M. (2025). Skeletal muscle fatigue in rats is more consistently related to increased inorganic phosphate concentration than acidosis. Acta Physiologica, 241, e70083. 10.1111/apha.70083 40686424 PMC12278345

[phy270871-bib-0043] Marôco, J. L. , Pinto, M. , Santa‐Clara, H. , Fernhall, B. , & Melo, X. (2022). Flow‐mediated slowing shows poor repeatability compared with flow‐mediated dilation in non‐invasive assessment of brachial artery endothelial function. PLoS One, 17, e0267287. 10.1371/journal.pone.0267287 35609038 PMC9129018

[phy270871-bib-0044] Marôco, J. L. , Silvestre, T. , Arrais, I. , Pinto, M. , Santa‐Clara, H. , Fernhall, B. , & Melo, X. (2023). Intra and inter‐rater repeatability of brachial artery ultrasound estimates of flow‐mediated slowing and flow‐mediated dilation. PLoS One, 18, e0287759. 10.1371/journal.pone.0287759 37379344 PMC10306196

[phy270871-bib-0045] Micklewright, D. , St Clair Gibson, A. , Gladwell, V. , & Al Salman, A. (2017). Development and validity of the rating‐of‐fatigue scale. Sports Medicine, 47, 2375–2393. 10.1007/s40279-017-0711-5 28283993 PMC5633636

[phy270871-bib-0046] Mori, K. , Nakaya, Y. , Sakamoto, S. , Hayabuchi, Y. , Matsuoka, S. , & Kuroda, Y. (1998). Lactate‐Induced Vascular Relaxation in Porcine Coronary Arteries is Mediated by Ca^2+^‐activated K^+^ Channels. 10.1006/jmcc.1997.0598 9515011

[phy270871-bib-0047] Muntner, P. , Shimbo, D. , Carey, R. M. , Charleston, J. B. , Gaillard, T. , Misra, S. , Myers, M. G. , Ogedegbe, G. , Schwartz, J. E. , Townsend, R. R. , Urbina, E. M. , Viera, A. J. , White, W. B. , & Wright, J. T. (2019). Measurement of blood pressure in humans: A scientific statement from the american heart association. Hypertension, 73, E35–E66. 10.1161/HYP.0000000000000087 30827125 PMC11409525

[phy270871-bib-0048] Padilla, J. , Harris, R. A. , Fly, A. D. , Rink, L. D. , & Wallace, J. P. (2006). A comparison between active‐ and reactive‐hyperaemia‐induced brachial artery vasodilation. Clinical Science, 110, 387–392. 10.1042/CS20050328 16356163

[phy270871-bib-0049] Priest, S. E. , Shenouda, N. , & MacDonald, M. J. (2018). Effect of sex, menstrual cycle phase, and monophasic oral contraceptive pill use on local and central arterial stiffness in young adults. American Journal of Physiology. Heart and Circulatory Physiology, 315, H357–H365. 10.1152/ajpheart.00039.2018 29677465 PMC6139630

[phy270871-bib-0050] Robergs, R. A. , McNulty, C. R. , Minett, G. M. , Holland, J. , & Trajano, G. (2018). Lactate, not lactic acid, is produced by cellular cytosolic energy catabolism. Physiology, 33, 10–12. 29212886 10.1152/physiol.00033.2017

[phy270871-bib-0051] Russ, D. W. , Kent‐Braun, J. A. , & Kent‐Braun, J. A. (2003). Sex differences in human skeletal muscle fatigue are eliminated under ischemic conditions. Journal of Applied Physiology (1985), 94, 2414–2422. 10.1152/japplphysiol 12562681

[phy270871-bib-0052] Russ, D. W. , Lanza, I. R. , Rothman, D. , & Kent‐Braun, J. A. (2005). Sex differences in glycolysis during brief, intense isometric contractions. Muscle & Nerve, 32, 647–655. 10.1002/mus.20396 16025523

[phy270871-bib-0053] Saltin, B. (2007). Exercise hyperaemia: Magnitude and aspects on regulation in humans. Journal of Physiology, 58, 819–823. 10.1113/jphysiol.2007.136309 PMC2277197 17640931

[phy270871-bib-0054] Saltin, B. , & Mortensen, S. P. (2012). Inefficient functional sympatholysis is an overlooked cause of malperfusion in contracting skeletal muscle. Journal of Physiology, 590, 6269–6275. 22988143 10.1113/jphysiol.2012.241026 PMC3533189

[phy270871-bib-0055] Shenouda, N. , Priest, S. E. , Rizzuto, V. I. , & MacDonald, M. J. (2018). Brachial artery endothelial function is stable across a menstrual and oral contraceptive pill cycle but lower in premenopausal women than in age‐matched men. American Journal of Physiology. Heart and Circulatory Physiology, 315, H366–H374. 10.1152/ajpheart.00102.2018 29727219

[phy270871-bib-0056] Siddiqi, A. K. , Ali, K. M. , Maniya, M. T. , Rashid, A. M. , Khatri, S. A. , Garcia, M. , Quintana, R. A. , & Naeem, M. (2024). The hidden epidemic: Hypertension‐related mortality surges amongst younger adults in the United States. Current Problems in Cardiology, 49, 102842. 39270766 10.1016/j.cpcardiol.2024.102842

[phy270871-bib-0057] Slattery, D. J. , Stuckless, T. J. R. , King, T. J. , & Pyke, K. E. (2016). Impaired handgrip exercise‐induced brachial artery flow‐mediated dilation in young obese males. Applied Physiology, Nutrition, and Metabolism, 41, 528–537. 10.1139/apnm-2015-0459 26985988

[phy270871-bib-0058] Smart, N. A. , Way, D. , Carlson, D. , Millar, P. , McGowan, C. , Swaine, I. , Baross, A. , Howden, R. , Ritti‐Dias, R. , Wiles, J. , Cornelissen, V. , Gordon, B. , Taylor, R. , & Bleile, B. (2019). Effects of isometric resistance training on resting blood pressure: Individual participant data meta‐analysis. Journal of Hypertension, 37, 1927–1938. 30889048 10.1097/HJH.0000000000002105 PMC6727950

[phy270871-bib-0059] Smith, J. R. , Koepp, K. E. , Berg, J. D. , Akinsanya, J. G. , & Olson, T. P. (2019). Influence of sex, menstrual cycle, and menopause status on the exercise Pressor reflex. Medicine and Science in Sports and Exercise, 51, 874–881. 10.1249/MSS.0000000000001877 30986812 PMC6467496

[phy270871-bib-0060] Szijgyarto, I. C. , King, T. J. , Ku, J. , Poitras, V. J. , Gurd, B. J. , & Pyke, K. E. (2013). The impact of acute mental stress on brachial artery flow‐mediated dilation differs when shear stress is elevated by reactive hyperemia versus handgrip exercise. Applied Physiology, Nutrition and Metabolism, 38, 498–506. 10.1139/apnm-2012-0328 23668756

[phy270871-bib-0061] Szijgyarto, I. C. , Poitras, V. J. , Gurd, B. J. , & Pyke, K. E. (2014). Acute psychological and physical stress transiently enhances brachial artery flow‐mediated dilation stimulated by exercise‐induced increases in shear stress. Applied Physiology, Nutrition, and Metabolism, 39, 927–936. 10.1139/apnm-2013-0384 24921439

[phy270871-bib-0062] Teixeira, A. L. , Garland, M. , Lee, J. B. , Nardone, M. , & Millar, P. J. (2022). Assessing functional sympatholysis during rhythmic handgrip exercise using Doppler ultrasound and near‐infrared spectroscopy: Sex differences and test‐retest reliability. American Journal of Physiology. Regulatory, Integrative and Comparative Physiology, 323, R810–R821. 10.1152/ajpregu.00123.2022 36189987

[phy270871-bib-0063] Thijssen, D. H. J. , Bruno, R. M. , van Mil, A. C. C. M. , Holder, S. M. , Faita, F. , Greyling, A. , Zock, P. L. , Taddei, S. , Deanfield, J. E. , Luscher, T. , Green, D. J. , & Ghiadoni, L. (2019). Expert consensus and evidence‐based recommendations for the assessment of flow‐mediated dilation in humans. European Heart Journal, 40, 2534–2547. 10.1093/eurheartj/ehz350 31211361

[phy270871-bib-0064] Thompson, B. C. , Fadia, T. , Pincivero, D. M. , Scheuermann, B. W. , & Scheuermann, B. W. (2007). Forearm blood flow responses to fatiguing isometric contractions in women and men. American Journal of Physiology. Heart and Circulatory Physiology, 293, 805–812. 10.1152/ajpheart.01136.2006.-Previous 17468333

[phy270871-bib-0065] Tremblay, J. C. , & Pyke, K. E. (2018). Flow‐mediated dilation stimulated by sustained increases in shear stress: A useful tool for assessing endothelial function in humans? American Journal of Physiology. Heart and Circulatory Physiology, 314, 508–520. 10.1152/ajpheart.00534.2017.-Investigations PMC5899264 29167121

[phy270871-bib-0066] Wan, J. J. , Qin, Z. , Wang, P. Y. , Sun, Y. , & Liu, X. (2017). Muscle fatigue: General understanding and treatment. Experimental and Molecular Medicine, 49, e384. 28983090 10.1038/emm.2017.194 PMC5668469

[phy270871-bib-0067] Williams, J. S. , Dunford, E. C. , Macdonald, M. J. , & West, M. S. (2020). Impact of the menstrual cycle on peripheral vascular function in premenopausal women: Systematic review & meta‐analysis. American Journal of Physiology. Heart and Circulatory Physiology, 319(6), H1327–H1337. 33064553 10.1152/ajpheart.00341.2020

[phy270871-bib-0068] Yu, A. , Giannone, T. , Scheffler, P. , Doonan, R. J. , Egiziano, G. , Gomez, Y. H. , Papaioannou, T. G. , & Daskalopoulou, S. S. (2014). The effect of oral contraceptive pills and the natural menstrual cYCLe on arterial stiffness and hemodynamICs (CYCLIC). Journal of Hypertension, 32, 100–107. 10.1097/HJH.0000000000000012 24326993

