# Supplementary material for: Brachial blood flow and pressure responses are unrelated to the greater isometric handgrip tolerance of females compared to males
Source: Physiol Rep. 2026 Apr 15;14(8):e70871. doi: 10.14814/phy2.70871 (PMC13083034; doi:10.14814/phy2.70871)
Supplement: Supplementary file 1 — Table S1. Brachial blood flow and mean pressure responses during and a 15‐s post‐fatiguing isometric handgrip presented as relative change to baseline. [file PHY2-14-e70871-s001.docx]

| **Supplemental Table 1**. Brachial blood flow and mean pressure responses during and a 15-s post-fatiguing isometric handgrip presented as relative change to baseline | | | | | | | | |
| --- | --- | --- | --- | --- | --- | --- | --- | --- |
|  | IHG─BAS | | Post5s─BAS | | Post10s─BAS | | Post15s─BAS | |
| Brachial blood flow, % | *p* | g | *p* | g | *p* | g | *p* | g |
|  | 0.415 | -0.31 | 0.324 | -0.90 | **0.021** | -0.90 | **0.019** | -0.92 |
| Female | 104 (79) | | 301 (152) | | 327 (148) | | 343 (144) | |
| Male | 132 (100) | | 373 (225) | | 491 (204) | | 503 (192) | |
| Brachial MAP, % | *p* | g | *p* | g | *p* | g | *p* | g |
|  | **0.025** | -0.90 | 0**.012** | -1.00 | 0.016 | -0.96 | **0.015** | -0.95 |
| Female | 20 (14) | | 9 (11) | | 2 (10) | | 1 (8) | |
| Male | 40 (27) | | 25 (18) | | 15 (13) | | 12 (11) | |
| Data presented as mean (SD). Each relative change presented was compared between sexes using Welch’s two-sample t-test, with the corresponding Hedges’ g effect size reported. Hedges’ g effect sizes were computed as the standardized mean difference (Female ─ Male), with more negative values indicating smaller increases in females. | | | | | | | | |
