# Supplementary material for: Brachial blood flow and pressure responses are unrelated to the greater isometric handgrip tolerance of females compared to males
Source: Physiol Rep. 2026 Apr 15;14(8):e70871. doi: 10.14814/phy2.70871 (PMC13083034; doi:10.14814/phy2.70871)
Supplement: Supplementary file 2 — Table S2. Linear mixed models for brachial blood flow, mean arterial pressure (MAP), and systolic blood pressure (SBP) controlled for brachial diameter, arm circumference, and maximum voluntary contraction (MVC). [file PHY2-14-e70871-s002.docx]

| **Supplemental Table 2.** Linear mixed models for brachial blood flow, mean arterial pressure (MAP), and systolic blood pressure (SBP) controlled for brachial diameter, arm circumference, and maximum voluntary contraction (MVC) | | | | | | | | | | | |
| --- | --- | --- | --- | --- | --- | --- | --- | --- | --- | --- | --- |
|  | df num | | df den | | F | *p* | | | Partial ω^2^ |  |  |
| **Brachial blood flow model** | | | | | | | | | |  |  |
| Sex | 1 | | 25 | | 0.109 | 0.744 | | | 0.00 |  |  |
| Time | 5 | | 137 | | 34.11 | **<0.001** | | | 0.53 |  |  |
| Sex*Time | 5 | | 133 | | 5.760 | **<0.001** | | | 0.15 |  |  |
| Covariates |  | |  | |  |  | | |  |  |  |
| bMAP | 1 | | 133 | | 0.366 | 0.547 | | | 0.00 |  |  |
| Brachial diameter | 1 | | 50 | | 18.55 | **<0.001** | | | 0.26 |  |  |
| Arm circumference | 1 | | 27 | | 1.840 | 0.187 | | | 0.03 |  |  |
| MVC | 1 | | 27 | | 0.119 | 0.733 | | | 0.00 |  |  |
| **Brachial MAP model** | | | | | | | | | | | |
| Sex | 1 | 26 | | 0.217 | | | 0.645 | 0.00 | | |  |
| Time | 5 | 137 | | 54.490 | | | **<0.001** | 0.65 | | |  |
| Sex*Time | 5 | 133 | | 4.647 | | | **<0.001** | 0.12 | | |  |
| Covariates |  |  | |  | | |  |  | | |  |
| Brachial diameter | 1 | 84 | | 0.226 | | | 0.635 | 0.00 | | |  |
| Arm circumference | 1 | 27 | | 0.588 | | | 0.450 | 0.00 | | |  |
| MVC | 1 | 27 | | 0.217 | | | 0.645 | 0.00 | | |  |
| **Brachial SBP model** | | | | | | | | | | |  |
| Sex | 1 | 27 | | 0.931 | | | 0.344 | 0.00 | | |  |
| Time | 5 | 137 | | 28.080 | | | **<0.001** | 0.49 | | |  |
| Sex*Time | 5 | 133 | | 2.826 | | | **0.018** | 0.06 | | |  |
| Covariates |  |  | |  | | |  |  | | |  |
| Brachial diameter | 1 | 84 | | 0.439 | | | 0.509 | 0.00 | | |  |
| Arm circumference | 1 | 27 | | 2.621 | | | 0.117 | 0.05 | | |  |
| MVC | 1 | 27 | | 5.254 | | | **0.030** | 0.13 | | |  |
